# 12 Months Persistent Immunogenicity after Hepatitis B Vaccination in Patients with Type 2 Diabetes and Immunogenicity of Revaccination in Non-Responders: An Open-Label Randomized Controlled Trial

**DOI:** 10.3390/vaccines9121407

**Published:** 2021-11-29

**Authors:** Bingfeng Han, Wu Liu, Juan Du, Hanyu Liu, Tianshuo Zhao, Shubo Yang, Shuai Wang, Sihui Zhang, Bei Liu, Yaqiong Liu, Fuqiang Cui

**Affiliations:** 1 Department of Epidemiology and Biostatistics, School of Public Health, Peking University, Beijing 100191, China; hanbingfeng@pku.edu.cn (B.H.); liuhanyuu@bjmu.edu.cn (H.L.); ztshuoshuo@163.com (T.Z.); zhangsihui@bjmu.edu.cn (S.Z.); 2 Jingyuan County Center for Disease Control and Prevention, Baiyin 730600, China; bysjyxjkzx1@163.com (W.L.); ysb13893070511@163.com (S.Y.); 3 Department of Laboratorial Science and Technology & Vaccine Research Center, School of Public Health, Peking University, Beijing 100191, China; juandu@bjmu.edu.cn (J.D.); 1916387057@bjmu.edu.cn (B.L.); liuyaqiong@bjmu.edu.cn (Y.L.); 4 Department of Infectious Diseases and Clinical Microbiology, Beijing Chao-Yang Hospital, Capital Medical University, Beijing 100020, China; wangpkq@163.com

**Keywords:** diabetes mellitus, hepatitis B vaccines, vaccination, persistent immunogenicity, revaccination

## Abstract

Background: In initial studies, the immunogenicity and safety of hepatitis B vaccines in patients with diabetes has been assessed in China. Methods: In six township health centers in Gansu Province, 232 diabetic patients and 77 healthy people were allocated to receive two 3-dose hepatitis B vaccines (Group D20SC 0-1-6; Group D20CHO 0-1-6; Group ND20SC 0-1-6). Participants were followed up at 12 months after being fully vaccinated. One dose of the vaccine was randomly administered to non-responders. Chi-square test was used to compare the differences in response rate between two groups. Results: The anti-HBs response rates of three groups decreased from 84.1%, 89.1% and 88.3% at one month to 64.6%, 79.8% and 71.4% at twelve months. There was no statistical difference in the immune response rates between Group D20SC 0-1-6 and Group ND20SC 0-1-6; however, that of Group D20CHO 0-1-6 was higher than that of Group D20SC 0-1-6. After revaccination, the geometric mean concentrations were 491.7 mIU/mL and 29.7 mIU/mL after using vaccines containing 60 μg and 20 μg HBsAg. Conclusions: At 12 months, immune response in diabetic patients were not significantly different from that in healthy people. Revaccination with one dose of hepatitis B vaccine containing 60 μg HBsAg for non-responders was more satisfactory.

## 1. Introduction

Hepatitis B virus (HBV) infection is a global public health threat with over 296 million people worldwide chronically infected and 820,000 hepatitis B-related deaths in 2019, and there are 1.5 million new infections each year [1]. In China, the prevalence of HBsAg was 5–6% with about 70 million HBsAg carriers [2], many of which were adults older than 20 years [3,4,5]. Based on a national serosurvey, only 13.8% of adults reported having received a hepatitis B vaccination [6], and many were anti-HBs negatives, indicating there were still many susceptible people of HBV infection.

China has the largest prevalence of diabetes in the world. The latest epidemiological study suggested that approximately 11% of the population had diabetes, with a significant proportion remaining undiagnosed [7]. Diabetic patients were considered to have a higher risk of HBV infection, and inadequate disinfection and cleaning of blood glucose monitors may lead to HBV transmission [8,9,10]. In 2017, hepatitis B vaccination of patients with diabetes was recommended by WHO for the first time [11]. In China, HBV prevention in children has been highly successful and increasingly effective for many years [12,13]. In 2019, a guideline published by Chinese Medicine Association recommended the hepatitis B vaccination for the diabetic population for the first time, but it did not become a national policy [14]. Hepatitis B vaccination in adults with diabetes has not received enough attention, and the safety, immunogenicity and immune persistence are still lacking evidence.

In the initial study, we conducted a phase IV, open-label, randomized, controlled study to evaluate the immunogenicity and safety of hepatitis B vaccination in patients with type 2 diabetes in China. We identified that the *Saccharomyces cerevisiae* recombinant and Chinese hamster ovary (CHO) cell recombinant hepatitis B vaccines contained 20 μg HBsAg, which can induce good immunogenicity one month after completing three doses of the vaccines, at months 0, 1, and 6. In addition, 9.5% of fully vaccinated diabetic participants were found to have no response [15]. Studies have shown that around 5–10% adults fail to respond or respond poorly to three doses of hepatitis B vaccine [16,17]. In China, the non-responding rate in adults after hepatitis B primary vaccination was around 4.7–14.2% [18,19,20]. Revaccination was the solution for non-responders of the vaccination.

In this study, fully vaccinated participants were followed up for 12 months, and no-responders were revaccinated with one dose of hepatitis B vaccine at random. We aimed to examine the 12-month persistent immunogenicity after hepatitis B vaccination in patients with type 2 diabetes and compare the immunogenicity of different revaccinations on non-responders.

## 2. Methods

### 2.1. Study Design

In the initial study, we did an open-label, randomized, controlled trial between January 2019 and August 2019 at six township health centers in Jingyuan County, Gansu Province, China (ChiCTR1800020190). All participants were randomly recruited. Eligible diabetic participants (1) were aged > 18 years; (2) were diagnosed with type 2 diabetes with accompanying medical records in health centers or hospitals (see the Supplementary File S1 for diagnostic criteria); (3) had three negative hepatitis B serological biomarkers (HBsAg, anti-HBs and anti-HBc); (4) had no hepatitis B vaccination before; and (5) provided informed consent. The healthy control group was also from the same six health centers. Eligible healthy controls (1) had at least four tests showing a normal fasting blood glucose level; (2) had three negative hepatitis B serological biomarkers (HBsAg, anti-HBs and anti-HBc); (3) had no hepatitis B vaccination before; and (4) provided informed consent. In addition, each of these controls was matched with one of the diabetic patients when they were recruited. The matching condition shall meet at least four of the following five items: (1) the control was the same sex as the diabetic patient; (2) the age difference between the diabetic patient and the control was between ±5 years; (3) the level of education was similar in the diabetic patient and the control; (4) the marital status was similar in the diabetic patient and the control; and (5) the occupation was similar in the diabetic patient and the control. Exclusion criteria were that (1) participants were pregnant during the study; (2) participants had physical disabilities or psychological diseases and were unable to complete the questionnaire.

Participants with diabetes were randomly assigned (1:1) to the two diabetic groups (Group D20SC 0-1-6 and Group D20CHO 0-1-6). Healthy participants were assigned to the healthy group (Group ND20SC 0-1-6). Contained 20 μg HBsAg of recombinant hepatitis B vaccine (*Saccharomyces cerevisiae* recombinant, aluminum vaccine adjuvants, Shenzhen Kangtai Biological Products Co., Ltd., Guangdong, China) was used according to a schedule of 0, 1 and 6 months in Group D20SC 0-1-6 and Group ND20SC 0-1-6. Contained 20 μg HBsAg of recombinant hepatitis B vaccine (CHO cell recombinant, aluminum vaccine adjuvants, NCPC Genetech Biotechnology Co., Ltd., Hebei, China) was used according to a schedule of 0, 1 and 6 months in Group D20CHO 0-1-6. Both vaccines have been approved for marketing and were the main vaccines available for Chinese adults.

We followed up all participants one month after being fully vaccinated and evaluated the immunogenicity and safety of different vaccines [15]. Participants were imputed as non-responders if their anti-HBs concentrations were <10 mIU/mL.

After the initial study, we followed up all 12 months after being fully vaccinated and determined the 12-month persistent immunogenicity, whose main outcome indicators were the proportion of responders (anti-HBs levels ≥ 10 mIU/mL) and high-level responders (anti-HBs levels ≥ 100 mIU/mL) [21,22]. We randomly administered one dose of *Saccharomyces cerevisiae* recombinant hepatitis B vaccine (containing 20 μg or 60 μg HBsAg, Shenzhen Kangtai Biological Products Co., Ltd., Guangdong, China) to non-responders and evaluated the immunogenicity one month after revaccination.

See Supplementary Figure S1 for a description of the study design, including baseline screening, routine vaccinations, and blood sampling timepoints.

### 2.2. Laboratory Assays

Blood samples were collected one month and 12 months after being fully vaccinated. Sera were stored and transported to the laboratory of Peking University School of Public Health (Beijing, China) for testing. The concentration of anti-HBs was quantitatively assessed via chemiluminescence microparticle immunoassay (CMIA) (reagents purchased from Abbott Ireland Diagnostics Division, Sligo, Ireland). The laboratory staff were masked to the various group assignments.

### 2.3. Ethical Approval

The protocol was approved by the Peking University Health Science Center Ethics Committee (IRB00001052), and the study was conducted according to the guidelines of the Declaration of Helsinki and Good Clinical Practice. Informed consent was obtained from all subjects involved before any study-related procedures were performed. All participants were granted the right to withdraw from the study without the need to provide a reason for their request. Information about all participants was handled with high levels of confidentiality and anonymity.

### 2.4. Statistical Analysis

PASS (Power Analysis and Sample Size, Version: 15.0.5, NCSS Statistical Software, Kaysville, UT, USA) was used to calculate the necessary sample size (see Supplementary File S2 for detailed calculation results of sample size).

Statistical Product and Service Solutions (SPSS, version 20.0, IBM, New York, NY, USA) was used for statistical analysis. Two analyses were performed in both per-protocol population and intention-to-vaccinate population. The anti-HBs immune response rates and high-level response rates were calculated in each group. The concentration of anti-HBs was transformed to a log (base 10) variable to compute the geometric mean concentrations (GMCs). Chi-square test was used to compare the differences in response rate or high-level response rate between two groups. The anti-HBs concentrations of responders, one month after being fully vaccinated, were divided into four segments: 10–100 mIU/mL, 100–1000 mIU/mL, 1000–10,000 mIU/mL, and >10,000 mIU/mL, respectively. Chi-square test of linear-by-linear association was used to evaluate the change in trend of HbsAb immune response rate 12 months after being fully vaccinated.

## 3. Results

### 3.1. Participants’ Characteristics at Baseline

**Figure 1:**
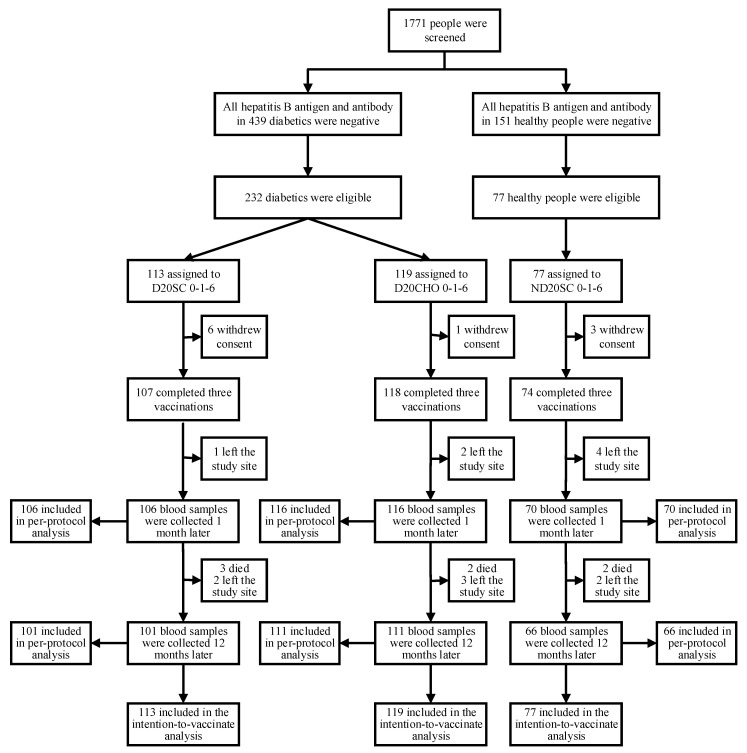
Participant flowchart in hepatitis B vaccine trial investigating the 12-month persistence of immunity in diabetic patients and healthy controls.

In the initial study, 113 diabetic patients were enrolled and randomly assigned to Group D20SC 0-1-6, and 119 were assigned to Group D20CHO 0-1-6. In total, 225 diabetic patients and 74 healthy people completed three vaccinations. Of these, 222 diabetic patients (106 in Group D20SC 0-1-6 and 116 in Group D20CHO 0-1-6) and 70 healthy people were followed-up one month after being fully vaccinated, and 212 diabetic patients (101 in Group D20SC 0-1-6 and 111 in Group D20CHO 0-1-6) and 66 healthy people were followed-up twelve months after being fully vaccinated (participant flowchart and the number of people included in both per-protocol and intention-to-vaccinate analyses are shown in Figure 1). The participants of this study were mainly women (61.0%) and people aged 50–60 years old (48.3%). Most of the participants were married (93.5%) and had a low education level (68.2% had primary school education or below). There was no difference in characteristics between the three groups one month after being fully vaccinated (Table 1).

D20SC 0-1-6: Diabetic group vaccinated with recombinant hepatitis B vaccine (20 μg HBsAg, *Saccharomyces cerevisiae* recombinant) according to the schedule of 0–1–6 months; D20CHO 0-1-6: Diabetic group vaccinated with recombinant hepatitis B vaccine (20 μg HBsAg, Chinese hamster ovary cells (CHO) recombinant) according to the schedule of 0–1–6 months; ND20SC 0-1-6: Control group vaccinated with recombinant hepatitis B vaccine (20 μg HBsAg, *Saccharomyces cerevisiae* recombinant) according to the schedule of 0–1–6 months.

### 3.2. Anti-HBs Immune Response Rate and High-Level Response Rate One and Twelve Months after Being Fully Vaccinated

In the intention-to-vaccinate population, the immune response rates (anti-HBs levels ≥ 10 mIU/mL) of Group D20SC 0-1-6, Group D20CHO 0-1-6 and Group ND20SC 0-1-6 decreased from 84.1%, 89.1% and 88.3% at one month to 64.6%, 79.8% and 71.4% at twelve months after being fully vaccinated, respectively. The high-level response rates (anti-HBs levels ≥ 100 mIU/mL) of those decreased from 70.8%, 78.2% and 77.9% at one month to 32.7%, 42.0% and 44.2% at twelve months after being fully vaccinated, respectively. Similar trends were also shown in the per-protocol population. The anti-HBs immune response rates of Group D20SC 0-1-6, Group D20CHO 0-1-6 and Group ND20SC 0-1-6 decreased from 89.6%, 91.4% and 97.1% at one month to 72.3%, 85.6% and 83.3% at twelve months after being fully vaccinated, respectively. The high-level response rates of those decreased from 75.5%, 80.2% and 85.7% at one month to 36.6%, 45.0% and 51.5% at twelve months after being fully vaccinated, respectively. The above decreases were statistically significant, except for the immune response rates of Group D20CHO 0-1-6 in the per-protocol analysis (Supplementary Table S1).

**Figure 2:**
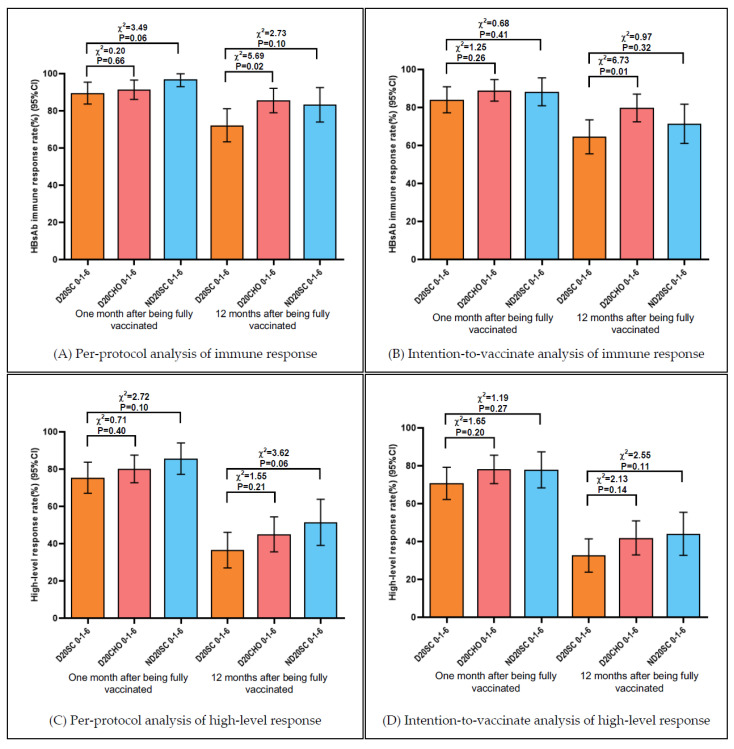
Immune response and high-level response at month 1 and 12 after vaccination in the intention-to-vaccinate and per-protocol analyses.

One month after being fully vaccinated, there was no statistical difference in the anti-HBs immune response rates and high-level response rates between three groups both in the intention-to-vaccinate population and per-protocol population. Statistical differences were still not found in the high-level response rates of those twelve months after being fully vaccinated. There was no statistical difference in the immune response rates between Group D20SC 0-1-6 and Group ND20SC 0-1-6; however, the immune response rate of Group D20CHO 0-1-6 twelve months after being fully vaccinated was higher than that of Group D20SC 0-1-6, and the difference was statistically significant (*p* = 0.01 in the intention-to-vaccinate population, and *p* = 0.02 in the per-protocol population) (Figure 2).

D20SC 0-1-6: Diabetic group vaccinated with recombinant hepatitis B vaccine (20 μg HBsAg, *Saccharomyces cerevisiae* recombinant) according to the schedule of 0–1–6 months; D20CHO 0-1-6: Diabetic group vaccinated with recombinant hepatitis B vaccine (20 μg HBsAg, Chinese hamster ovary cells (CHO) recombinant) according to the schedule of 0–1–6 months; ND20SC 0-1-6: Control group vaccinated with recombinant hepatitis B vaccine (20 μg HBsAg, *Saccharomyces cerevisiae* recombinant) according to the schedule of 0–1–6 months.

### 3.3. Maintenance of Anti-HBs Concentration after Twelve Months

In two diabetic groups, the proportion of participants whose anti-HBs remained positive twelve months after being fully vaccinated increased with the increase of initial concentration one month after being fully vaccinated (35.7% when initial anti-HBs concentration was 10–100 mIU/mL, 84.1% when initial anti-HBs concentration was 100–1000 mIU/mL, 95.2% when initial anti-HBs concentration was 1000–10,000 mIU/mL, 96.4% when initial anti-HBs concentration was > 10,000 mIU/mL). A similar trend also existed in the healthy control group (50.0% when initial anti-HBs concentration was 10–100 mIU/mL, 75.0% when initial anti-HBs concentration was 100–1000 mIU/mL, 87.5% when initial anti-HBs concentration was 1000–10,000 mIU/mL, 100.0% when initial anti-HBs concentration was >10,000 mIU/mL). Both the two trends were statistically significant (*p* < 0.01 in diabetic groups and healthy control group). However, the differences in these proportions maintained between the diabetic groups and the control group were not found in any segment (Table 2).

### 3.4. Response after Revaccination of Non-Responders in Diabetic Patients

Twenty-one diabetic patients did not respond to hepatitis B vaccination in the initial study. There was no statistical difference in the characteristics of non-responders after vaccination in the per-protocol population (Supplementary Table S2). Nineteen participants (two withdrew consent) were randomly divided into two groups (ten in Group SV60, and nine in Group SV20), and vaccinated with one dose of hepatitis B vaccine containing 60 μg or 20 μg HBsAg. A month later, the anti-HBs immune response rate in Group SV60 was 100%, and that in Group SV20 was 66.7%. The GMC in Group SV60 was 491.7 mIU/mL, while that in Group SV20 was 29.7 mIU/mL (Supplementary Table S3). In terms of concentration distribution, the anti-HBs concentration of eight participants (80.0%) exceeded 100 mIU/mL after revaccination in Group SV60, but that of seven participants (77.7%) was less than 100 mIU/mL after revaccination in Group SV20. There was significant difference in antibody concentration distribution between these two groups (*p* = 0.048) (Table 3).

## 4. Discussion

In the initial study, compared with healthy controls, two 3-dose hepatitis B vaccines containing 20 μg HBsAg can induce good immunogenicity (response rates exceeded 80%) without additional risk in Chinese diabetic populations one month after being fully vaccinated [15]. In this study, 12-month persistent immunogenicity induced by them was satisfactory in patients with type 2 diabetes, especially for the CHO recombinant hepatitis B vaccine. For non-responders, revaccination with one-dose hepatitis B vaccine containing 60 μg HBsAg could induce a higher anti-HBs concentration than that with the vaccine containing 20 μg HBsAg.

The immune response of the hepatitis B vaccine in the two diabetic groups was not significantly lower than that in the healthy control within 12 months. In particular, CHO cell recombinant hepatitis B vaccine showed a stronger ability to maintain immune response in patients with diabetes. CHO cell recombinant hepatitis B vaccine showed comparable effectiveness with yeast derived ones [23], and it could induce good and stable long-term efficacy [24,25,26]. Good humoral and cellular immunity effects were identified by several studies [18,27], but there is no clear evidence of its mechanism. A possible explanation was that the HBsAg expressed from CHO cells had a higher glycosylation, which could induce a higher humoral immunity [18]. More studies on the immunogenicity mechanism of hepatitis B vaccine are still needed. A twelve-month follow-up study supported the two domestic vaccines that could be applied to Chinese diabetic populations. However, the anti-HBs immune response rates and high-level response rates of three groups showed a downward trend in 12 months. In particular, the latter decreased by about 50%, which was consistent with the trend of antibody attenuation. This trend could also be observed in newborns [28], adolescents [29], and healthy young people [30] and adults [31]. Several factors have been suggested to be associated with the response to hepatitis B vaccination, such as older ages [32], use of medication [33], BMI ≥ 25, smoking, and concomitant disease [34]. This study emphasized the importance of hepatitis B vaccination, especially in patients with diabetes, based on the little difference of response rate with healthy controls. A meta-analysis showed that the immunization program schedule at months 0, 1, and 12 was also worth choosing when the schedule at months 0, 1, and 6 could not be completed [35].

In this segmented study of anti-HBs concentration, we did not find any statistical difference in the maintenance of anti-HBs concentration between patients with diabetes and healthy controls. It indicated that the trend of anti-HBs attenuation was consistent between diabetic patients and healthy people. The proportion of participants whose anti-HBs remained positive twelve months after being fully vaccinated increased with the increase of initial concentration one month after being fully vaccinated, which meant that higher anti-HBs concentrations are associated with longer protection duration. It was considered to be consistent with previous studies [31,36,37]. Moreover, this study suggests that subjects with a low anti-HBs concentration at the primary response should be carefully followed up.

For immunity failure, revaccination on non-responders may confer further protection against HBV infection [38,39]. After revaccination, both the immune response and the high-level response, as well as GMC of anti-HBs concentration induced by hepatitis B vaccine containing 60 μg HBsAg, were higher than those of vaccines containing 20 μg HBsAg. The former was recommended for low or non-responders [40,41]. This study verified that hepatitis B vaccine containing 60 μg HBsAg was more satisfactory in non-responders with diabetes. Immune failure may be related to several endogenous factors, such as failure in antigen presentation or the stimulation of T helper cells [42], and direct involvement of the HLA-DRB1 gene [43]. One-dose revaccination for non-responders appeared economical and convenient, but the long-term immunogenicity after the revaccination still needs to be observed.

The results of this study support the public health significance of hepatitis B vaccination for diabetic patients in China. A report showed that diabetic patients had nearly double the risk of developing acute HBV infections as healthy adults [8]. Based on the high prevalence of diabetes in China, the potential risk of hepatitis B outbreaks was high. However, more than 85% of the immune response and good 12-month immune persistence of hepatitis B vaccination made diabetic patients well protected, reducing a lot of potential hepatitis B cases, and even many cases and deaths of cirrhosis and liver cancer.

There were some potential study limitations in this study. Firstly, there were few non-responders in this study, so the sample size was not enough to support statistical tests. In addition, only one dose of the vaccine was revaccinated in this study, rather than three doses of the vaccine according to the schedule. Secondly, the follow-up time of responders and participants receiving revaccination was still short, which could not fully explain the immune persistence. The cohort of the diabetes group and the control group in this study will be maintained for a long time, and it is expected to obtain more detailed information on immune persistence in a few years.

## 5. Conclusions

In summary, at 12 months after being fully vaccinated, the anti-HBs immune response and the high-level response in diabetic patients were not significantly different from that in healthy people. Patients with diabetes who had been vaccinated with CHO recombinant hepatitis B vaccine had a higher response rate at 12 months after being fully vaccinated in this study. It showed a downward trend during the first year after the primary vaccination, but high-level response dropped quickly. Revaccination with one dose of hepatitis B vaccine containing 60 μg HBsAg for non-responders was more satisfactory.

## Figures and Tables

**Table 1:** Characteristics of participants in diabetic and healthy groups one month after being fully vaccinated.

	D20SC 0-1-6	D20CHO 0-1-6	ND20SC 0-1-6	*χ* ^2^	*p*
Sex				3.08	0.21
Female	70 (66.0)	71 (61.2)	37 (52.9)		
Male	36 (34.0)	45 (38.8)	33 (47.1)		
Age (years)				7.88	0.10
≤50	18 (17.0)	21 (18.1)	21 (30.0)		
50–60	52 (49.1)	54 (46.6)	35 (50.0)		
>60	36 (34.0)	41 (35.3)	14 (20.0)		
Education				6.71	0.15
Senior high school or above	5 (4.7)	9 (7.8)	9 (12.9)		
Junior high school	23 (21.7)	26 (22.4)	21 (30.0)		
Primary school or below	78 (73.6)	81 (69.8)	40 (57.1)		
Marriage				2.40	0.30
Married	96 (90.6)	110 (94.8)	67 (95.7)		
Unmarried	10 (9.4)	6 (5.2)	3 (4.3)		
Occupation				2.13	0.35
Farmer	105 (99.1)	112 (96.6)	67 (95.7)		
Others	1 (0.9)	4 (3.4)	3 (4.3)		
BMI (Mean ± Standard deviation)	23.7 (3.1)	22.8 (2.8)	23.9 (3.5)	2.50 ^a^	0.08 ^b^
Duration of diabetes diagnosis (years)					
≤2	13 (12.6)	25 (24.3)		5.77	0.12
2–4	34 (33.0)	29 (28.2)			
4–7	23 (22.3)	32 (31.1)			
>7	36 (35.0)	30 (29.1)			
Total	106 (100.0)	116 (100.0)	70 (100.0)		

^a^: F value of analysis of variance (ANOVA); ^b^: *p* value of ANOVA. D20SC 0-1-6: Diabetic group vaccinated with recombinant hepatitis B vaccine (20 μg HBsAg, *Saccharomyces cerevisiae* recombinant) according to the schedule of 0–1–6 months; D20CHO 0-1-6: Diabetic group vaccinated with recombinant hepatitis B vaccine (20 μg HBsAg, Chinese hamster ovary cells (CHO) recombinant) according to the schedule of 0–1–6 months; ND20SC 0-1-6: Control group vaccinated with recombinant hepatitis B vaccine (20 μg HBsAg, *Saccharomyces cerevisiae* recombinant) according to the schedule of 0–1–6 months.

**Table 2:** The ability of different anti-HBs concentrations to maintain positive twelve months after being fully vaccinated.

Anti-HBs Concentration (mIU/mL)	Diabetic Groups	Control Group	*χ*^2^ *	*p*
	Responders 12 Months after Being Fully Vaccinated/Initial Responders (%)	Responders 12 Months after Being Fully Vaccinated/Initial Responders (%)		
10–100	10/28 (35.7)	4/8 (50.0)	0.10	0.75
100–1000	69/82 (84.1)	18/24 (75.0)	0.53	0.47
1000–10,000	60/63 (95.2)	21/24 (87.5)	0.64	0.42
>10,000	27/28 (96.4)	12/12 (100.0)	0.00	1.00
*χ*^2^ #	35.58	8.44		
*p*	<0.01	<0.01		

* Continuity correction; # linear by linear association.

**Table 3:** Distribution of anti-HBs concentration after revaccination of non-responders in diabetic patients.

Anti-HBs Concentration (mIU/mL)	Group SV60	Group SV20	*χ* ^2^	*p*
	N. (%)	N. (%)		
<10	0 (0)	3 (33.3)	7.92	0.048
10–100	2 (20.0)	4 (44.4)		
100–1000	5 (50.0)	2 (22.2)		
1000–10,000	3 (30.0)	0 (0)		
Total	10 (100)	9 (100)		

SV60: Revaccinated with one dose of recombinant hepatitis B vaccine (60 μg HBsAg, *Saccharomyces cerevisiae* recombinant); SV20: revaccinated with one dose of recombinant hepatitis B vaccine (20 μg HBsAg, *Saccharomyces cerevisiae* recombinant).

## Data Availability

All data will be provided on reasonable demand. These were stored on password protected computers at the Department of Laboratorial Science and Technology and Vaccine Research Center, School of Public Health, Peking University. Readers who wish to gain access to the data can write to the corresponding author.

## Supplementary Materials

The following are available online at https://www.mdpi.com/article/10.3390/vaccines9121407/s1. Supplementary File S1: Criteria for the diagnosis of diabetes; Supplementary Figure S1: Study design including baseline screening, routine vaccinations, and blood sampling timepoints; Supplementary File S2: Sample size calculated by PASS; Supplementary Table S1: Response at months 1 and 12 after being fully vaccinated for participants in the per-protocol analysis and the intention-to-vaccinate analysis; Supplementary Table S2: Characteristics of non-responders after vaccination in the per-protocol population; Supplementary Table S3: Response after revaccination of non-responders in diabetic patients.
