# Supplementary material for: 12 Months Persistent Immunogenicity after Hepatitis B Vaccination in Patients with Type 2 Diabetes and Immunogenicity of Revaccination in Non-Responders: An Open-Label Randomized Controlled Trial
Source: Vaccines (Basel). 2021 Nov 29;9(12):1407. doi: 10.3390/vaccines9121407 (PMC8705985; doi:10.3390/vaccines9121407)
Supplement: Supplementary file 1 [file vaccines-09-01407-s001.zip › vaccines-1450102-supplementary.pdf]

Supplementary File S1 Criteria for the diagnosis of diabetes

According to the Chinese guidelines for the prevention and treatment of type 2 diabetes (2017 Edition), if the subjects meet the following conditions, they will be defined as type 2 diabetes in this study.

First, according to the medical records to determine the object to meet at least one of the following three conditions:

(1) In a patient with classic symptoms of hyperglycemia or hyperglycemic crisis, a random plasma glucose ≥11.1 mmol/L.
(2) Fasting plasma glucose (FPG) ≥7.0 mmol/L. Fasting is defined as no caloric intake for at least 8 hours.
(3) 2-h plasma glucose ≥ 11.1 mmol/L during oral glucose tolerance test (OGTT). OGTT should use a glucose load containing the equivalent of 75 g anhydrous glucose dissolved in water.

Second, for the subjects that met the criteria in the previous step, whether HbA_{1c} has been detected in the medical records is queried. If not, venous blood will be collected and HbA_{1c} will be detected. When HbA_{1c} ≥6.3%, the subjects are defined as type 2 diabetes.

Note: In the methods and results of this study, “people with diabetes” refer to “people with type 2 diabetes”.

Reference:

Chinese Diabetes Society. Chinese guidelines for the prevention and treatment of type 2 diabetes (2017 Edition). Chinese Journal of Diabetes Mellitus. 2018,10(1):4-67. DOI:10.3760/cma.j.issn.1674-5809.2018.01.003.

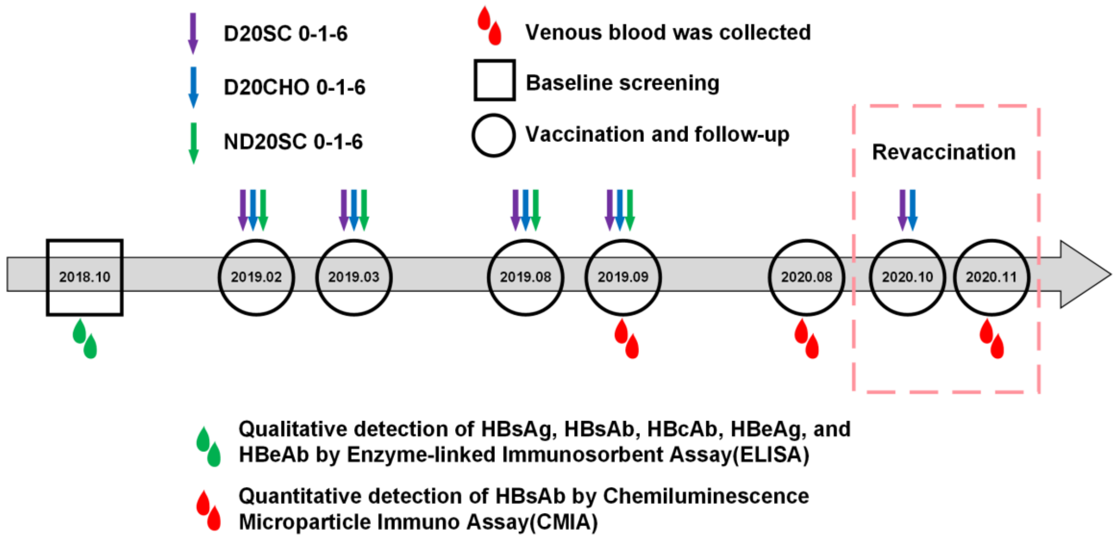

Note:

✧ D20SC 0-1-6: Diabetic group vaccinated with recombinant hepatitis B vaccine (20μg HBsAg, Saccharomyces cerevisiae recombinant) according to the schedule of 0-1-6 month;

✧ D20CHO 0-1-6: Diabetic group vaccinated with recombinant hepatitis B vaccine (20μg HBsAg, Chinese hamster ovary cells (CHO) recombinant) according to the schedule of 0-1-6 month;

✧ ND20SC 0-1-6: Control group vaccinated with recombinant hepatitis B vaccine (20μg HBsAg, Saccharomyces cerevisiae recombinant) according to the schedule of 0-1-6 month.

Supplementary Figure S1 Study design including baseline screening, routine vaccinations, and blood sampling timepoints

Supplementary File S2 Sample size calculated by PASS

Design Tab

Solve For	Sample Size
Power Calculation Method	Normal Approximation
Alternative Hypothesis	One-Sided
Test Type	Z-Test (Unpooled)
Power	0.8
Alpha	0.05
Group Allocation	Equal ($N_1 = N_2$)
Input Type	Differences
D_1 (Difference|H1 = P1-P2)	-0.15
P_2 (Group 2 Proportion)	0.95

Numeric Results for Testing Two Proportions using the Z-Test with Pooled Variance

H_0: $P_1 - P_2 = D_1 \geq 0$ vs. H_1: $P_1 - P_2 = D_1 < 0$.

Target Power	Actual Power*	Diabetic group (N_1)	Healthy group (N_2)	Seroconversion rate in the healthy group (P_2)	Differences (D_1)	Alpha
0.80	0.80592	58	58	0.95	-0.15	0.05

* Power was computed using the normal approximation method.

Report Definitions

Target Power is the desired power value (or values) entered in the procedure. Power is the probability of rejecting a false null hypothesis.

Actual Power is the power obtained in this scenario. Because N_1 and N_2 are discrete, this value is often (slightly) larger than the target power.

N_1 and N_2 are the number of items sampled from each population.

P_1 is the proportion for treatment or experimental group at which power and sample size calculations are made.

P_2 is the proportion for healthy group.

D_1 is the difference $P_1 - P_2$ assumed for power and sample size calculations.

Alpha is the probability of rejecting a true null hypothesis.

Summary Statements

Group sample sizes of 58 in diabetic group and 58 in healthy group achieve 80.592% power to detect a difference between the group proportions of -15%. The proportion in diabetic group is assumed to be 95% under the null hypothesis and 80% under the alternative hypothesis. The proportion in healthy group is 95%. The test statistic used is the one-sided Z-Test with unpooled variance. The significance level of the test is 0.05.

Dropout-Inflated Sample Size

Dropout Rate	Sample Size		Dropout-Inflated Enrollment		Expected Number of Dropouts	
	N_1	N_2	N_1'	N_2'	D_1'	D_2'
20%	58	58	73	73	15	15

Definitions

Dropout Rate (DR) is the percentage of subjects (or items) that are expected to be lost at random during the course of the study and for whom no response data will be collected (i.e. will be treated as “missing”).

N_1 and N_2 are the evaluable sample sizes at which power is computed. If N1 and N2 subjects are evaluated out of the N1' and N2' subjects that are enrolled in the study, the design will achieve the stated power.

N_1' and N_2' are the number of subjects that should be enrolled in the study in order to end up with N_1 and N_2 evaluable subjects, based on the assumed dropout rate. After solving for N1 and N_2, N_1' and N_2' are calculated by inflating N_1 and N_2 using the formulas $N_1' = N1 / (1 - DR)$ and $N_2' = N_2 / (1 - DR)$, with N_1' and N_2' always rounded up. (See Chow, S.C., Shao, J., and Wang, H. (2008) pages 39-40.)

D_1' and D_2' are the expected number of dropouts. $D_1' = N_1' - N_1$, and $D_2' = N_2' - N_2$.

References

[1] Chow, S.C., Shao, J., Wang, H. Sample Size Calculations in Clinical Research. Second Edition. Chapman & Hall/CRC. Boca Raton, Florida. 2008.

[2] D’Agostino, R.B., Chase, W., Belanger, A. The Appropriateness of Some Common Procedures for Testing the Equality of Two Independent Binomial Populations. The American Statistician, 1988, 42(3):198-202.

[3] Fleiss, J. L., Levin, B., Paik, M.C. Statistical Methods for Rates and Proportions. Third Edition. John Wiley & Sons. New York. 2003.

[4] Lachin, John M. Biostatistical Methods. John Wiley & Sons. New York. 2000.

[5] Machin, D., Campbell, M., Fayers, P., Pinol, A. Sample Size Tables for Clinical Studies, 2nd Edition. Blackwell Science. Malden, Mass. 1997.

[6] Ryan, Thomas P. Sample Size Determination and Power. John Wiley & Sons. Hoboken, New Jersey. 2013.

Supplementary Table S1 Response at months 1 and 12 after being fully vaccinated for participants in the Per-protocol analysis and Intention-to-vaccinate analysis

Time of assessment*	Per-protocol analysis, No. of Responders/No. Undergoing Testing (%)			Intention-to-vaccinate analysis, No. of Responders/No. of participants (%)		
	D20SC 0-1-6 (n=106)	D20CHO 0-1-6 (n=116)	ND20SC 0-1-6 (n=70)	D20SC 0-1-6 (n=113)	D20CHO 0-1-6 (n=119)	ND20SC 0-1-6 (n=77)
One month after being fully vaccinated						
Response	95/106(89.6)	106/116(91.4)	68/70(97.1)	95/113(84.1)	106/119(89.1)	68/77(88.3)
High-level response	80/106(75.5)	93/116(80.2)	60/70(85.7)	80/113(70.8)	93/119(78.2)	60/77(77.9)
12 months after being fully vaccinated						
Response	73/101(72.3)	95/111(85.6)	55/66(83.3)	73/113(64.6)	95/119(79.8)	55/77(71.4)
High-level response	37/101(36.6)	50/111(45.0)	34/66(51.5)	37/113(32.7)	50/119(42.0)	34/77(44.2)
Statistical difference						
Response	χ^2=6.27, P=0.01	χ^2=1.88, P=0.17	χ^2=7.49, P=0.01	χ^2=11.23, P<0.01	χ^2=3.87, P=0.049	χ^2=6.83, P=0.01
High-level response	χ^2=30.75, P<0.01	χ^2=30.03, P<0.01	χ^2=18.61, P<0.01	χ^2=32.77, P<0.01	χ^2=32.39, P<0.01	χ^2=18.46, P<0.01

* Response indicates hepatitis B surface antibody (HBsAb) concentrations of 10 mIU/mL or greater; High-level response indicates HBsAb concentrations of 100 mIU/mL or greater

- ✧ D20SC 0-1-6: Diabetic group vaccinated with recombinant hepatitis B vaccine (20μg HBsAg, Saccharomyces cerevisiae recombinant) according to the schedule of 0-1-6 month;
- ✧ D20CHO 0-1-6: Diabetic group vaccinated with recombinant hepatitis B vaccine (20μg HBsAg, Chinese hamster ovary cells (CHO) recombinant) according to the schedule of 0-1-6 month;
- ✧ ND20SC 0-1-6: Control group vaccinated with recombinant hepatitis B vaccine (20μg HBsAg, Saccharomyces cerevisiae recombinant) according to the schedule of 0-1-6 month.

Supplementary Table S2 Characteristics of non-responders after vaccination in the per-protocol population

	D20SC 0-1-6				D20CHO 0-1-6				ND20SC 0-1-6			
	N	Number of non-responders (%)	χ^2	*P*	N	Number of non-responders (%)	χ^2	*P*	N	Number of non-responders (%)	χ^2	*P*
Sex												
Female	70	7(10.0)	0.03	0.86	71	6(8.5)	0.01	0.93	37	2(5.4)	0.41	0.52
Male	36	4(11.1)			45	4(8.9)			33	0(0)		
Age(years)												
≤50	18	1(5.6)	2.38	0.30	21	1(4.8)	0.91	0.63	21	1(4.8)	0.69	0.71
50-60	52	4(7.7)			54	6(11.1)			35	1(2.9)		
>60	36	6(16.7)			41	3(7.3)			14	0(0)		
Education												
Senior high school and above	5	0(0)	1.96	0.38	9	0(0)	2.51	0.29	9	0(0)	4.80	0.09
Junior high school	23	4(17.4)			26	4(15.4)			21	2(9.5)		
Primary school and below	78	7(9.0)			81	6(7.4)			40	0(0)		
Marriage												
Married	96	11(11.5)	1.28	0.26	110	8(7.3)	2.15	0.14	67	1(1.5)	2.15	0.14
Unmarried	10	0(0)			6	2(33.3)			3	1(33.3)		
Diabetic duration(years)												
≤2	13	1(7.7)	1.57	0.67	25	3(12.0)	4.40	0.22				
2-4	34	3(8.8)			29	1(3.4)						
4-7	23	4(17.4)			32	5(15.6)						
>7	36	3(8.3)			30	1(3.3)						
**Total**	**106**	**11(10.4)**			**116**	**10(8.6)**			**70**	**2(2.9)**		

✧ D20SC 0-1-6: Diabetic group vaccinated with recombinant hepatitis B vaccine (20μg HBsAg, Saccharomyces cerevisiae recombinant) according to the schedule of 0-1-6 month;

✧ D20CHO 0-1-6: Diabetic group vaccinated with recombinant hepatitis B vaccine (20μg HBsAg, Chinese hamster ovary cells (CHO) recombinant) according to the schedule of 0-1-6 month;

✧ ND20SC 0-1-6: Control group vaccinated with recombinant hepatitis B vaccine (20μg HBsAg, Saccharomyces cerevisiae recombinant) according to the schedule of 0-1-6 month.

Supplementary Table S3 Response after revaccination of non-responders in diabetic patients

Group	Non responders in the initial study	Responders after revaccination (%)	GMC after revaccination(mIU/mL)
SV60	10	10(100.0)	491.7
SV20	9	6(66.7)	29.7

- ✧ SV60: Revaccinated with one dose of recombinant hepatitis B vaccine (60μg HBsAg, Saccharomyces cerevisiae recombinant);
- ✧ SV20: Revaccinated with one dose of recombinant hepatitis B vaccine (20μg HBsAg, Saccharomyces cerevisiae recombinant)
